# Distribution of intestinal parasites of baboons (*Papio anubis*) and warthogs (*Phacochoerus aethiopicus*) at the Mole National Park, Ghana

**DOI:** 10.1002/vms3.335

**Published:** 2020-08-09

**Authors:** John A. Larbi, Stephen Akyeampong, Seth Offei Addo, Kwaku Brako Dakwa, Kwadwo Boampong, Bright Opoku‐Nketiah

**Affiliations:** ^1^ Department of Theoretical and Applied Biology Kwame Nkrumah University of Science and Technology Kumasi Ghana; ^2^ Parasitology Department Noguchi Memorial Institute for Medical Research University of Ghana Accra Ghana; ^3^ Department of Conservation, Biology and Entomology University of Cape Coast Cape Coast Ghana

**Keywords:** baboons, intestinal parasites, mole, prevalence, warthogs

## Abstract

The identification of intestinal parasite of baboons (*Papio anubis*) and warthogs (*Phacochoerus aethiopicus*) was undertaken at the Mole National Park, Ghana. The main objective of the study was to determine the types and prevalence of intestinal parasites in baboons and warthogs in the Mole National Park. A total of nineteen (19) and twenty‐three (23) samples were collected from the baboons and warthogs, respectively, and examined using the direct saline smear and formol‐ether concentration technique for the identification of cysts, eggs and larvae of parasites. The survey showed that 94.74% of the baboon samples examined was infected with at least one parasite, whereas that of the warthogs showed 95.65% prevalence. A total of seven (7) and eight (8) different parasites were identified in baboon and warthog faecal samples, respectively. *Strongyloides* sp. had the highest prevalence in baboons (84.21%) and warthogs (78.26%). The second prevalent parasite identified was *Ascaris* sp. in the baboons (31.58%) and warthogs (30.43%). The results showed a high level of multiparasitism in these wild animals and an increased risk of zoonotic transmission which may result from interaction with inhabitants of the park community.

## Graphical abstract

The identification of intestinal parasite of baboons (*Papio anubis*) and warthogs (*Phacochoerus aethiopicus*) was undertaken at the Mole National Park, Ghana. Strongyloides had the highest prevalence in baboons (84.21%) and warthogs (78.26%). The results showed a high level of multiparasitism in these wild animals and an increased risk of zoonotic transmission which may result from interaction with inhabitants of the park community.

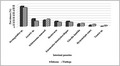

## 1 INTRODUCTION

Infections caused by gastrointestinal parasites have become widespread and a growing threat to public health (Ezenwa, 2003). These parasites are capable of causing infections in humans and non‐human primates (Jourdan, Lamberton, Fenwick, & Addiss, 2018). It has become essential to understand the nature of zoonotic disease transmission especially in areas where humans and animals interact frequently (Bowden & Drake, 2013). Anthropogenic habitats serve as a highly prone area for disease transmission as changes force humans and non‐human primates to have persistent contact (Nunn, Gillespie, Wich, & Marshall, 2016). The situation at the Mole National park is one that allows the interaction of free‐ranging animals with the surrounding inhabitants. Such an environment will most likely promote the transmission of zoonotic parasites. Baboons and warthogs represent the group of animals which can be found interacting the most with inhabitants of Mole. It has become common to find them using human garbage as a steady source of food, hence these animals pay daily visits to the homes of inhabitants. It has been observed that baboons can harbour and serve as a source of intestinal parasites which are pathogenic to humans (Mafuyai, Barshep, Audu, Kumbak, & Ojobe, 2013). More so, an earlier study recorded a high rate of intestinal parasites in baboons sampled from the Mole National park some of which are known to infect humans (Ryan et al., 2012). The ecosystem of Mole is a woodland savanna with riparian forests (Bowell & Ansah, 1994) which presents an environment that can influence the diversity of parasites harboured by animals (Eggert, Rasner, & Woodruff, 2002). Thus, it is important to identify the intestinal parasites within the animal population to ascertain their role in parasite transmission and the risk of human infections.

## 2 MATERIALS AND METHODS

### 2.1 Study area

The National Park is the largest and the most prestigious, protected Park in Ghana located in the West Gonja District of the Savannah Region of Ghana. It covers approximately 4,480 km^2^ of land and is located within latitude 9°12′–10°06′N, and longitude 1°25′–2°17′W. The park represents a fairly undisturbed guinea savannah ecosystem that is dominated by open savannah woodland with very rich flora and fauna. The park is home to a number of wildlife including mammals, birds, reptiles, amphibians, insects and a vast majority of plant species (www.britanica.com).

### 2.2 Sample collection

Faecal samples were taken from both baboons (19) and warthogs (23) within the Park in March 2017. The sampling was performed close to the living quarters of inhabitants within the park. The animals were trailed and as they dropped their faeces it was collected while still fresh using a disposable spatula. Caution was taken to ensure samples were taken from different animal hosts. The collected faecal samples were preserved in 10% formalin in well‐labelled airtight sample containers and transported to the laboratory for examination.

### 2.3 Sample analysis

A qualitative examination of the samples with a microscope was conducted using the saline wet mount method (thin saline smear) to identify eggs of the intestinal parasites, with help from bench aids in the laboratory.

The concentrated technique (Formol‐ether concentration technique) was also carried out for further identification of eggs as the thin saline smear may not detect parasites if the number of parasite ova or cyst in the stool specimen is low (Cheesbrough, 2006). *Strongyloides* eggs are usually ellipsoid, with a length of about 40–85 μm and a thin wall containing a larva whereas *Ascaris* eggs often appear rounded, thick‐shelled with an external mamillated layer that is often stained brown by bile and have a length of about 45–75 µm. However, Hookworm eggs have a thin shell, appear colourless and have a dimension of 60–75 µm by 35–40 µm.

## 3 RESULTS

**FIGURE 1:**
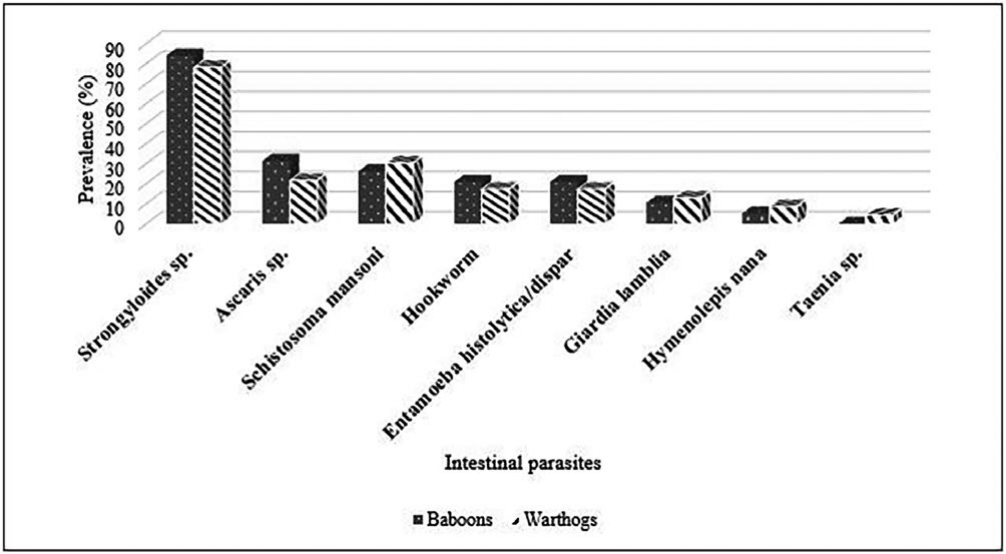
Comparison of parasites in Baboons (*Papio anubis*) and Warthogs (*Phacochoerus aethiopicus*)

A total of nineteen faecal samples of baboons (*Papio anubis*) in the Mole National Park were collected and examined for intestinal parasites. Eighteen of the samples, representing 94.74% were infected with one or more intestinal parasite species. Seven different species of parasites were encountered, of which five were helminths, and two were protozoan parasites. *Strongyloides* sp. (84.21%) was found in 16 of the samples, representing the highest prevalence (Figure 1). Other parasites identified were *Ascaris* sp. (31.58%), Hookworm (21.05%)*, Giardia lamblia* (10.53%)*, Schistosoma mansoni* (26.32%), *Hymenolepis nana* (5.26%) and *Entamoeba histolytica/dispar* (21.05%).

Differences in prevalence regarding the parasite species was not significant (*p* = .538) at CI 95% using the chi‐square test of proportion.

### 3.1 Multiparasitism in baboons (*Papio anubis*)

Ten samples from baboons contained two parasite species representing 52.63%, whereas five baboons had three different species of parasites (26.32%).

### 3.2 Prevalence of intestinal parasites in warthogs (*Phacochoerus aethiopicus*)

The warthogs found in the Mole National Park are of the species *Phacochoerus aethiopicus*. Out of the 23 stool samples examined from warthogs in the Mole National Park, 22 samples were infected with one or more intestinal parasites representing a prevalence of 95.65% (Figure 1). A total of eight species of parasites were identified; Six helminths and two protozoan parasite species. With a prevalence of 78.26%, *Strongyloides* was the most encountered parasite and occurred in eighteen of the samples. Other parasites found in the samples were *Ascaris* (30.43%) and *Schistosoma mansoni* (21.74%). The parasite that occurred least was *Taenia spp*. with a prevalence of 4.35% (Figure 1).

### 3.3 Multiparasitism in warthogs (*Phacochoerus aethiopicus*)

Fifteen warthogs had two parasites, representing 65.22%, two warthogs had three parasites (8.69%), one warthog had four parasites and four had one parasite (4.35%).

### 3.4 Comparison of parasites in baboons and warthogs

The intestinal parasites encountered in baboons were morphologically similar to the parasites encountered in the warthogs. The prevalence of the parasites, however, differed among the two animals (Table 1). There was a higher prevalence of *Strongyloides, Ascaris,* Hookworm and *Entamoeba histolytica/dispar in* baboons than warthogs. However, the prevalence of *Schistosoma mansoni, Giardia lamblia* and *Hymenolepis nana* were higher in the warthogs. *Taenia* sp. occurred in warthogs and not in baboons. (Figure 1).

**TABLE 1:** Prevalence of intestinal parasites in baboons and warthogs at the Mole National Park

Parasite	Number of Baboons infected (% prevalence)	Number of Warthogs infected (% prevalence)
Hookworm	4 (21.05)	4 (17.39)
*Strongyloides* sp.	16 (84.21)	18 (78.26)
*Hymenolepis nana*	1 (5.26)	2 (8.70)
*Entamoeba histolytica/dispar*	4 (21.05)	4 (17.39)
*Giardia lamblia*	2(10.53)	3 (13.04)
*Ascaris* sp.	6 (31.58)	5 (21.74)
*Schistosoma mansoni*	5 (26.32)	7 (30.43)
*Taenia* sp.	0 (0)	1 (4.35)

There was no significant difference in the proportion of parasites between the two animals.

## 4 DISCUSSION

Behavioural activities such as grooming, foraging as well as individual and intergroup associations may influence the spread of parasitic infections among primates (Tiddi, Pfoh, & Agostini, 2019) and potentially to other animals in close proximity. The faecal samples for this study were collected from the sampled animals close to the residence of workers of the park. It was noticed that the baboons and warthogs, in particular, come close and interact with the people.

The detection of high infection rates of intestinal parasites in the faecal samples is an important finding for public health as baboons are zoonotic agents constituting a risk during interactions with humans and other animals. The results of this study are similar to that obtained from sampled *Papio anubis* in different localities in Ethiopia, which demonstrated the presence of different intestinal parasites including *Strongyloides* sp. (37.3%), *Schistosoma mansoni* (20.3%) and *Entamoeba histolytica/dispar* (16.9%) (Legesse & Erko, 2004). The high prevalence of *Strongyloides* sp. in baboons sampled for this study also correlates with that of baboons in West Bugwe Forest Reserve, Uganda, where the prevalence rate was 60.7% (Ocaido, Dranzoa, & Cheli, 2003).

*Strongyloides* has a two‐way transmission route. Transmission is either by the intake of food and water contaminated with faeces containing the infective stage or by infective filariform larvae penetrating the skin (Greaves, Coggle, Pollard, Aliyu, & Moore, 2013). This intestinal threadworm is unique in primates because only females can be parasitic, and they pass eggs or larvae into the faeces (Thamsborg, Ketzis, Horii, & Matthews, 2017). Autoinfection is also very common with *Strongyloides* and this enables untreated infections to persist and account for high numbers (Page, Judd, & Bradbury, 2018). The observed high prevalence of *Strongyloides* in the study area could be due to the unhygienic sanitary conditions in some areas, especially around the human habitations. Although the general prevalence of intestinal parasites in *Papio anubis* in the Mole National Park was high, the prevalence of individual parasites was on the whole low. The findings from this study is consistent with research in baboons (*Papio hamadryas*) in an area of hot, dry climatic conditions where the prevalence of gastrointestinal parasites was low (Ghandour, Zahid, Banaja, Kamal, & Bouq, 1995), with a high prevalence of parasites in areas of mild, cool climatic conditions.

As with this study, baboons are mostly infected with helminths than protozoans and this is in accordance with the more common helminth populations seen from baboons living in savannah conditions (Appleton & Brain, 1995). It was also observed that multiparasitism is common among the baboons in the Mole National Park. This might be due to immunosuppression caused by the presence of high numbers of *Strongyloides* sp. (George, 1998). However, detecting parasites such as Hookworms, *Entamoeba histolytica*, *Giardia lamblia* and *Ascaris* sp. suggest potential zoonotic transmission (Mafuyai et al., 2013). Thus, humans living in close proximity may be at a high risk of infection hence the need for preventive measures.

Warthogs are diurnal and spend most of their time looking for food. They wallow in ditches and refuse dumps in search of food and are therefore prone to infestations by many intestinal parasites. *Strongyloides* was the parasite that was found to infect most of the warthogs. It recorded a high prevalence of 78.26% which could be the result of cross‐contamination between the warthogs and the baboons or other wildlife as these animals interact a lot within their habitat and also search in the human habitat for food. Any of them could have fed on grass and leaves contaminated with faeces from the baboons which are infected heavily with *Strongyloides* sp. as well as other parasites. The relatively low prevalence of some of the parasites, hookworm and *Taenia* sp. could be attributed to several factors such as the intake of plant parts by the warthogs which could significantly reduce the intestinal parasite population. It is believed that some leaves consumed by these animals contain anthelminthic substances that prevent the establishment and multiplication of some intestinal parasites (Engel, 2002).

The baboons and warthogs at the Mole National Park seemed to be infected with similar parasites although molecular methods will need to be used to confirm. Observation of their behaviour showed that these animals interact, found close to human neighbourhoods and therefore there was an increased likelihood of cross‐contamination and infection. Baboons, however, recorded a higher prevalence of most of the parasites than the warthogs. The warthogs move around freely in the community and feed on the domestic wastes around human habitation in the park. The warthogs also go close to the toilet facilities in the community where they feed on grass. Compared to the baboons, the warthogs appeared closer to inhabitants (humans) and therefore pose a serious threat in terms of zoonotic disease transmission. Most of the parasites encountered are also parasites of humans and may infect the human population as well as other animals close to the community. Further investigations will, therefore, be required to fully determine the risk of zoonotic transmission and formulate control strategies to prevent the spread of infections.

## 5 CONCLUSIONS

There is a heavy infestation of baboons and warthogs at the Mole National Park with parasites that could possibly infect humans. *Strongyloides w*as the most prevalent intestinal parasite in both animals studied. It is therefore concluded that intestinal parasites occur at high prevalence in the baboons and warthogs at the Mole National Park. The animals may, therefore, serve as agents for the zoonotic transfer of some parasites, however, this needs to be investigated.

## CONFLICTS OF INTEREST

The authors declare there are no conflicts of interest in this study.

## AUTHOR CONTRIBUTION

**John Asiedu Larbi:** Conceptualization; Data curation; Investigation; Methodology; Project administration; Supervision; Writing‐original draft; Writing‐review & editing. **Stephen Akyeampong:** Conceptualization; Data curation; Investigation; Methodology; Project administration; Supervision; Writing‐review & editing. **Seth Offei Addo:** Data curation; Investigation; Methodology; Writing‐review & editing. **Kwaku Brako Dakwa:** Writing‐review & editing. **Kwadwo Boampong:** Data curation; Investigation; Writing‐review & editing. **Bright Opoku‐Nketiah:** Data curation; Investigation; Writing‐review & editing.

### PEER REVIEW

The peer review history for this article is available at https://publons.com/publon/10.1002/vms3.335.
